# Dyslipidemia is associated with monocyte lipid metabolic reprogramming in Guillain–Barré syndrome

**DOI:** 10.3389/fneur.2026.1797582

**Published:** 2026-04-13

**Authors:** Yuan Chen, Shuanghong Sun, Feihong Jia, Meng Li, Haining Wang, Jihe Song, Hongping Chen, Di Zhong

**Affiliations:** Department of Neurology, First Affiliated Hospital, Harbin Medical University, Harbin, Heilongjiang, China

**Keywords:** CEBPB, Guillain–Barré syndrome, lipid metabolism, monocytes reprogramming, single-cell RNA sequencing

## Abstract

GBS (Guillain–Barré syndrome) is an acute immune-mediated peripheral neuropathy whose pathological mechanisms remain incompletely understood. Our study aimed to explore the intrinsic link and potential mechanisms between circulating dyslipidemia and immune metabolic remodeling of monocytes in GBS. Clinical data from 163 patients diagnosed with classic GBS and 169 healthy controls were analyzed to examine the correlations between their lipid profiles and immune cell features. Leveraging our team’s previously published single-cell RNA sequencing (scRNA-seq) dataset for GBS, we characterized the subpopulations, functional features, and differentiation trajectories of peripheral blood monocytes. Furthermore, through transcriptional regulatory network analysis, we identified key transcription factors governing these processes. Compared to healthy controls, GBS patients displayed a distinct dyslipidemia characterized by elevated triglycerides (TG), very low-density lipoprotein (VLDL), and residual cholesterol (RC), but decreased high-density lipoprotein (HDL) and apolipoprotein A1 (APOA1). This lipid profile coincided with higher monocyte counts in peripheral blood, suggesting a synergistic relationship. The scRNA-seq analysis results indicate that the composition of peripheral blood monocyte subsets in GBS patients undergoes fundamental remodeling. This is characterized by a significant expansion of CD14^+^CD163^+^ monocytes lipid metabolism-active monocytes and pro-inflammatory CD14^+^CD169^+^ monocytes, while the proportion of classical monocytes sharply decreases. Pseudo-time trajectory analysis revealed a differentiation pathway from classical monocytes to CD14^+^CD163^+^ monocytes via CD14^+^CD169^+^ and intermediate monocytes. This pathway was characterized by an initial increase followed by a decrease in pro-inflammatory activity, coupled with a progressive enhancement in lipid metabolism activity. Transcriptional network analysis identified CEBPB as a core transcription factor potentially associated with the phenotypic conversion of monocyte subsets, likely mediated by synergistic regulation of genes involved in lipid metabolism and cell differentiation. In summary, GBS is characterized by synergistic dyslipidemia and monocyte remodeling. The pathological signature involves lipid metabolic reprogramming in the CD14^+^CD163^+^ monocytes and pro-inflammatory phenotypes in the CD14^+^CD169^+^ monocytes. The transcription factor CEBPB is associated with this phenotypic conversion by regulating lipid metabolism and differentiation genes, revealing molecular targets for precise GBS diagnosis and therapy.

## 1 Introduction

GBS is an immune-mediated acute polyradiculoneuropathy clinically characterized by quadriplegia, areflexia, and paresthesia. GBS has a global annual incidence of approximately 1.1 to 1.8 cases per 100,000 people (1). The overall incidence of GBS in China is relatively low, with an estimated annual rate of approximately 0.698 cases per 100,000 population (2). It is widely accepted that infections, such as with *Campylobacter jejuni*, are common triggers for GBS. Through molecular mimicry, such infections can trigger the production of autoantibodies targeting gangliosides in peripheral nerves, thereby activating the complement system and inducing demyelination or axonal damage, which ultimately leads to neurological dysfunction (3). Clinicians commonly utilize cerebrospinal fluid protein-cell dissociation testing, nerve conduction studies, and antibody detection as auxiliary diagnostic methods. However, these approaches have limitations: in early stages, cerebrospinal fluid protein-cell dissociation, nerve conduction velocity, and wave amplitude may not yet show typical abnormalities (4, 5); furthermore, some GBS patients remain without detectable known specific antibodies (6, 7). At present, the most effective therapeutic interventions are considered to be plasma exchange and intravenous immunoglobulin infusion (8). Nevertheless, despite the administration of standard treatment, approximately 7% of patients succumb to the disease, with a substantial number of survivors suffering from permanent disabilities (9).

Monocytes originate from hematopoietic stem cells in the bone marrow and primarily circulate in the bloodstream. They are broadly classified into classical, non-classical, and intermediate subtypes. Classical monocytes (CD14^+^CD16^−^ cMonos) are the predominant subset driving inflammatory responses and can differentiate into either inflammatory macrophages or tumor-associated macrophages. Intermediate monocytes (CD14^+^CD16^+^ iMonos) exhibit potent antigen-presenting and pro-inflammatory functions and are frequently associated with chronic inflammatory conditions. Non-classical monocytes (CD14^−^CD16^+^ ncMonos) patrol the vascular lumen, where they contribute to endothelial homeostasis, promote inflammation resolution, and help suppress tumor metastasis (10). Notably, dyslipidemia significantly impacts monocyte function and phenotype. For example, elevated triglycerides promote lipid accumulation within monocytes, activate pro-inflammatory signaling pathways, and upregulate the expression of inflammatory factors such as CD11c (11); high LDL remodels lipid metabolism and gene expression in hematopoietic stem cells in the bone marrow, driving their differentiation into pro-inflammatory monocytes and thereby fueling systemic inflammation (12). Moreover, mild oxidation of apolipoprotein B (APOB) generates bioactive oxidized phospholipids. These phospholipids activate platelet-activating factor receptors on the surface of monocytes, thereby promoting monocyte chemotaxis, enhancing adhesion to endothelial cells, and triggering the release of pro-inflammatory factors (13). On the other hand, APOA1, the major structural protein of HDL, shows a significant negative correlation with the expression of pro-inflammatory adhesion molecules (such as integrins CD11b, CD11c, and CD29) on the surface of monocytes. This suggests that APOA1 may exert anti-inflammatory effects by suppressing the expression of monocyte adhesion molecules (14). Previous clinical studies have demonstrated that patients with GBS often exhibit a pro-inflammatory lipid profile, characterized by elevated levels of APOB, RC, and TG, along with decreased levels of anti-inflammatory APOA1 and HDL (15). Further investigation has revealed a positive correlation between remnant cholesterol levels and the severity of GBS. The underlying mechanism may involve the activation of the TLR2/TLR4-NF-κB signaling pathway in monocytes, which in turn promotes inflammatory responses (16). Collectively, these findings suggest that dysregulated lipid metabolism may contribute to the pathophysiology of GBS by modulating monocyte function.

Based on this, our study integrates clinical retrospective research and single-cell transcriptome analysis. On one hand, it validates the dyslipidemia profile in patients with GBS; on the other hand, it conducts an in-depth analysis of the peripheral blood monocyte subpopulation remodeling, metabolic reprogramming, and potential transcriptional regulatory networks, thereby clarifying the intrinsic link between dyslipidemia and monocyte dysfunction. By exploring clues related to the immunometabolic mechanisms in GBS, our study provides a reference for the development of future targeted diagnostic and therapeutic strategies.

## 2 Methods

### 2.1 GBS patients and healthy controls

This study enrolled 163 classic GBS patients from the First Affiliated Hospital of Harbin Medical University, between February 2020 and January 2025. GBS diagnoses were reviewed and confirmed based on the Guidelines for the Diagnosis and Treatment of Guillain–Barré Syndrome in China issued in 2024. The inclusion criteria for GBS patients were as follows: (1) Age ≥ 18 years; (2) Confirmed diagnosis of classic GBS based on clinical and electrophysiological criteria, with subtypes including Acute Inflammatory Demyelinating Polyneuropathy, Acute Motor Axonal Neuropathy, and Acute Motor-Sensory Axonal Neuropathy. The exclusion criteria encompassed all GBS variants, namely Miller-Fisher syndrome, Bickerstaff brainstem encephalitis, pure sensory variant, pharyngeal-cervical-brachial variant, paraplegic variant, bilateral facial palsy with sensory disturbance variant, and autonomic variant. The healthy controls (matched by sex and age) comprised 169 subjects who underwent routine physical examinations. These individuals had no history of autoimmune or neurological disorders, nor any record of infection or vaccination within the preceding month.

### 2.2 Clinical data collection

We collected demographic and clinical baseline data (gender, age, and histories of diabetes, hypertension, and alcohol use) from all study subjects. The laboratory parameters included white blood cell count (WBC), neutrophil count (NEUT), lymphocyte count (LYMPH), monocyte count (MONO), total cholesterol (CHOL), TG, HDL, low-density lipoprotein cholesterol (LDL), VLDL, APOB, APOA1, and lipoprotein(a) (Lpa).

### 2.3 Data processing of the scRNA-seq

This study utilized previously published peripheral blood scRNA-seq data from our team, which have been deposited in the National Omics Data Encyclopedia under project accession numbers OEP002315 and OEP002701 (17). The dataset includes samples from 3 acute-phase GBS patients (within 14 days of symptom onset) and 3 healthy controls. Data analysis was performed using R studio (v4.3.1) and packages including Seurat (v4.3.0.1), Harmony (v1.2.3), and Single R (v2.2.0). First, cells expressing fewer than 50 genes, genes expressed in fewer than 3 cells, and cells with >25% mitochondrial gene content were filtered out, yielding 36,703 high-quality cells and 21,126 genes. Gene expression was normalized using the LogNormalize method. Subsequently, 1,500 highly variable genes were selected for principal component analysis The top 10 principal components were determined based on an elbow plot and visualized via t-SNE. Following initial automated cell annotation, manual verification and refinement were carried out using classical marker genes to ensure accurate cell type labeling.

### 2.4 Identification of monocyte subpopulations

All monocytes were isolated based on cell annotation for further analysis. Unsupervised clustering was conducted using the Louvain algorithm at a resolution of 0.2, based on the top 20 Harmony-corrected principal components. The results were visualized with UMAP. Differentially expressed genes were identified by the FindAllMarkers function with thresholds of |log₂FC| > 0.25. Based on established definitions of monocyte subsets (10), we categorized monocytes as follows: CD14^+^CD16^−^ cMonos, characterized by high expression of CD14 and LYZ; CD14^+^CD16^+^ iMonos, marked by high expression of CD14 and FCGR3A; and CD14^−^CD16^+^ ncMonos, defined by high expression of FCGR3A and CX3CR1. Furthermore, based on previous studies defining monocyte functional subpopulations: CD14^+^CD163^+^ monocytes (CD14^+^CD163^+^ Monos) exhibit high expression of both CD14 and CD163, and are recognized for their anti-inflammatory or regulatory potential (18, 19). CD14^+^CD169^+^ monocytes (CD14^+^CD169^+^ Monos) are characterized by high expression of CD14 and CD169 (SIGLEC1). These monocytes have potent antigen uptake and presentation capabilities and may participate in interferon-mediated immune activation (20, 21). Following the manual curation and removal of non-target cells (dendritic cells and macrophages) from the auto-clustered data, subpopulations were precisely annotated.

### 2.5 Enrichment analysis

Integrated gene sets were constructed based on the HALLMARK, Kyoto Encyclopedia of Genes and Genomes (KEGG), and REACTOME databases. Gene set enrichment analysis (GSEA) was performed using the fgsea package (v1.26.0) on the differentially expressed gene lists (sorted in descending order by log_2_FC) for each monocyte subset. The top 5 pathways with the highest normalized enrichment scores values for each subpopulation were visualized as heatmaps. For the CD14^+^CD163^+^ Monos subpopulation, Gene Ontology (GO) functional enrichment analysis was performed using the clusterProfiler package (v4.8.3), covering biological processes (BP), Cellular Component (CC), and Molecular Function (MF). Core enrichment results were visualized using bubble plots, with lipid metabolism-related GO terms highlighted. Gene–pathway associations were illustrated using chord diagrams generated with the circlize package (v0.4.16).

### 2.6 Cell trajectory analysis

Pseudotime trajectories for monocyte subpopulations were constructed using the Monocle3 package (v1.4.26). Classical monocytes (CD14^+^CD16^−^ cMonos) were designated as the trajectory origin, as they constitute the largest proportion (approximately 85%) in healthy controls and are widely recognized as the primary precursors for monocyte differentiation (22).

### 2.7 Pathway activity score

Pathways related to lipid metabolism, pro-inflammatory response, and anti-inflammatory response were selected from the three databases mentioned above. The AUCell package (v1.22.0) was used to calculate the activity scores for each cell across these three pathways. These pathway activity scores were mapped onto the pseudotime trajectories constructed by Monocle3 to analyze their dynamic changes during the differentiation process. Additionally, FeaturePlot visualized the spatial distribution of pro-inflammatory and anti-inflammatory pathway activities on the UMAP plot and analyzed their colocalization.

### 2.8 Transcription factor regulatory network

The FindMarkers function was used to identify differentially expressed genes (DEGs) between GBS patients and controls across all monocytes. DEGs were filtered based on the thresholds of |log₂FC| > 0.25. Lipid metabolism (LM)-related and cell differentiation (CD)-related gene sets were obtained from the three databases mentioned above. Subsequently, the DEGs were intersected with each of these two gene sets to obtain LM-related and CD-related DEG subsets. Finally, these two subsets were combined to generate the final target gene set. For each gene in the set, we queried the Cistrome Data Browser^1^ to obtain the top 10 predicted transcription factors (TFs). The occurrence frequency of all predicted TFs was calculated. TFs that were predicted for more than 60% of the target genes were identified as core TFs. Finally, a regulatory network depicting the relationships between the core TFs and their target genes was constructed and visualized.

### 2.9 Validation using the GSE31014 dataset

Download the independent dataset GSE31014 (containing peripheral blood leukocytes bulk RNA-seq data from 7 GBS patients and 7 healthy controls) from the GEO database. First, we used the xCell online tool^2^ to estimate the proportions of various immune cells in the samples. We additionally deconvoluted the proportions of CD14^+^CD163^+^ Monos and CD14^+^CD169^+^ Monos in GSE31014 using CIBERSORTx with our scRNA-seq-derived signature. Second, differential expression analysis between groups was performed on this data using the limma package (v3.56.2), with screening criteria set as |log₂FC| > 0.5. KEGG pathway enrichment analysis was conducted on the obtained differentially expressed genes, and the results were visualized using bubble plots. Finally, the expression of monocyte marker genes and core differentially expressed genes identified in the single-cell analysis was validated in this dataset via the Wilcoxon rank-sum test, with results displayed using box plots and heatmaps.

### 2.10 Statistical analyses

Statistical analyses were conducted in RStudio. Continuous variables were assessed for normality using the Shapiro–Wilk test. Normally distributed data with homogeneous variance are presented as mean ± SD and compared by independent samples *t*-test; otherwise, data are presented as median (IQR) and compared by Wilcoxon rank-sum test. Categorical variables are presented as frequency (%), with comparisons by chi-square or Fisher’s exact test. Associations between immune cells and blood lipids were assessed using Spearman’s rank correlation. For single-cell data, differential gene expression and pathway activity were compared between groups using the Wilcoxon rank-sum test, with False Discovery Rate (FDR) adjustment via the Benjamini-Hochberg method. Statistical significance was set at *p* < 0.05 (or FDR < 0.05).

## 3 Results

### 3.1 Differences in immune cells and blood lipid profiles between GBS patients and controls

**Figure 1:**
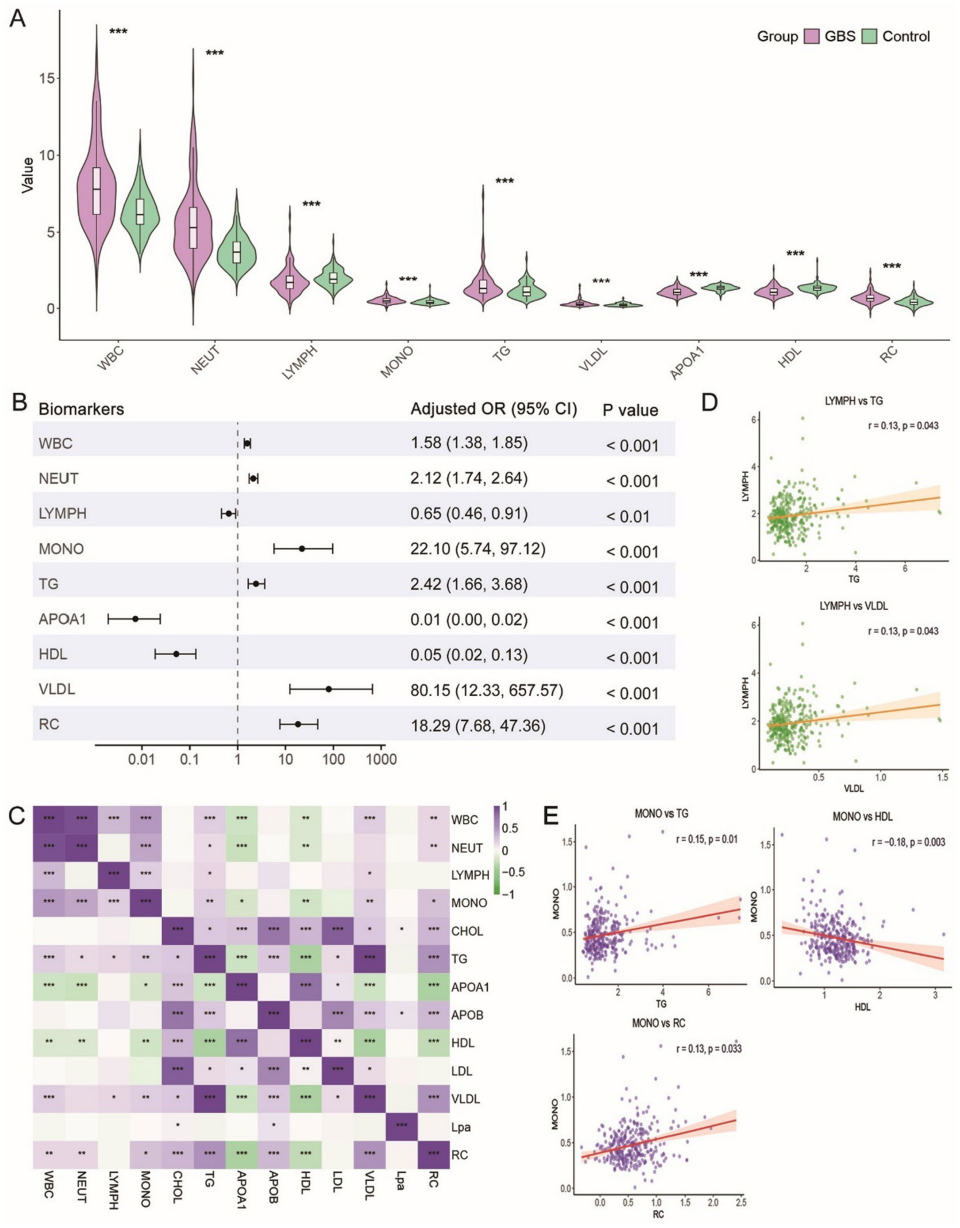
Differential and correlational analysis of immune cells and lipids in GBS and controls. **(A)** Violin plots showing immune cell counts and selected circulating lipid levels in GBS patients versus controls. GBS patients exhibited significantly higher monocyte counts, TG, and VLDL levels compared to controls, whereas APOA1 and HDL levels were significantly lower. **(B)** Multivariate logistic regression analysis of the independent association between immune/lipid biomarkers and GBS. **(C)** Correlation heatmap between immune cells and circulating lipid markers. Purple indicates a positive correlation; green indicates a negative correlation. **(D)** Scatter plot showing the correlation between LYMPH and TG as well as VLDL. **(E)** Scatter plot showing the correlation between MONO and TG, VLDL, APOA1, HDL, and RC. * *p* < 0.05; ** *p* < 0.01; *** *p* < 0.001.

Our study recruited 163 GBS patients and 169 healthy controls, with no significant differences detected in CHOL and APOB levels between the two cohorts. However, the proportion of individuals with diabetes and alcohol consumption history differed significantly between GBS patients and healthy controls (*p* < 0.05). Further biochemical and hematological analyses revealed that, relative to healthy controls, GBS patients had significantly higher circulating levels of WBC, NEUT, MONO, TG, VLDL, and RC, along with pronounced reductions in LYMPH counts, HDL, and APOA1 levels (*p* < 0.001) (Table 1; Figure 1A).

**Table 1:** Clinical and laboratory characteristics in patients with GBS and controls.

Characteristics	GBS (*n* = 163)	Control (*n* = 169)	*P*
Age, y	55 (42–66)	53 (42–62)	0.199
Male, *n* (%)	95 (56.2)	96 (58.9)	0.658
Diabetes, *n* (%)	18 (11)	6 (3.6)	0.015
Hypertension, *n* (%)	23 (13.6)	30 (18.4)	0.294
Alcohol, *n* (%)	17 (10.4)	3 (1.8)	0.002
WBC, ×10^9^/L	7.79 (6.15–9.19)	6.15 (5.46–7.16)	<0.001
NEUT, ×10^9^/L	5.29 (3.96–6.61)	3.70 (3.00–4.38)	<0.001
LYMPH, ×10^9^/L	1.68 (1.29–2.16)	1.94 (1.63–2.36)	<0.001
MONO, ×10^9^/L	0.49 (0.37–0.64)	0.39 (0.31–0.51)	<0.001
CHOL, mmol/L	4.54 (3.93–5.10)	4.60 (4.22–4.97)	0.499
TG, mmol/L	1.29 (0.99–1.88)	1.05 (0.81–1.41)	<0.001
APOA1, g/L	1.03 (0.89–1.24)	1.33 (1.23–1.44)	<0.001
APOB, g/L	0.92 (0.76–1.07)	0.90 (0.81–1.01)	0.887
HDL, mmol/L	1.04 (0.88–1.26)	1.32 (1.17–1.45)	<0.001
LDL, mmol/L	2.73 (2.30–3.31)	2.87 (2.53–3.20)	0.164
VLDL, mmol/L	0.26 (0.20–0.38)	0.21 (0.16–0.28)	<0.001
Lpa, (mg/L)	138.30 (74.51–295.24)	117.00 (59.73–243.70)	0.09
RC, mmol/L	0.65 (0.48–0.85)	0.40 (0.25–0.58)	<0.001

Data presented as median (Q1–Q3) or percentage.

To verify whether the observed differences in immune and lipid parameters were independently associated with GBS, multivariate logistic regression analyses were performed with adjustment for age, gender, diabetes, and alcohol consumption. Each biomarker was analyzed in a separate model to avoid multicollinearity. After adjustment, most immune and lipid parameters remained significantly associated with GBS. Specifically, WBC, NEUT, MONO, TG, VLDL, and RC were independently and positively associated with GBS, while LYMPH, HDL, and APOA1 were independently and negatively associated with GBS (*p* < 0.01) (Figure 1B).

Correlation analysis further revealed a statistically significant covariate network between immune cell subsets and circulating lipid markers (Figure 1C). Although the individual correlation coefficients were relatively modest (|r| ranging from 0.13 to 0.18), a consistent pattern was observed. Specifically, WBC, NEUT, and MONO levels were positively correlated with elevated TG, VLDL, and RC, and negatively correlated with decreased APOA1 and HDL levels. Conversely, lower lymphocyte counts were associated with higher TG and VLDL levels (*p* < 0.05, Figures 1D,E). These findings suggest that in GBS patients, systemic inflammatory activation and circulating lipid dysregulation are not independent events, but rather co-occur in a population-level synergy.

### 3.2 Single-cell analysis reveals immune cell remodeling in the peripheral blood of GBS patients

**Figure 2:**
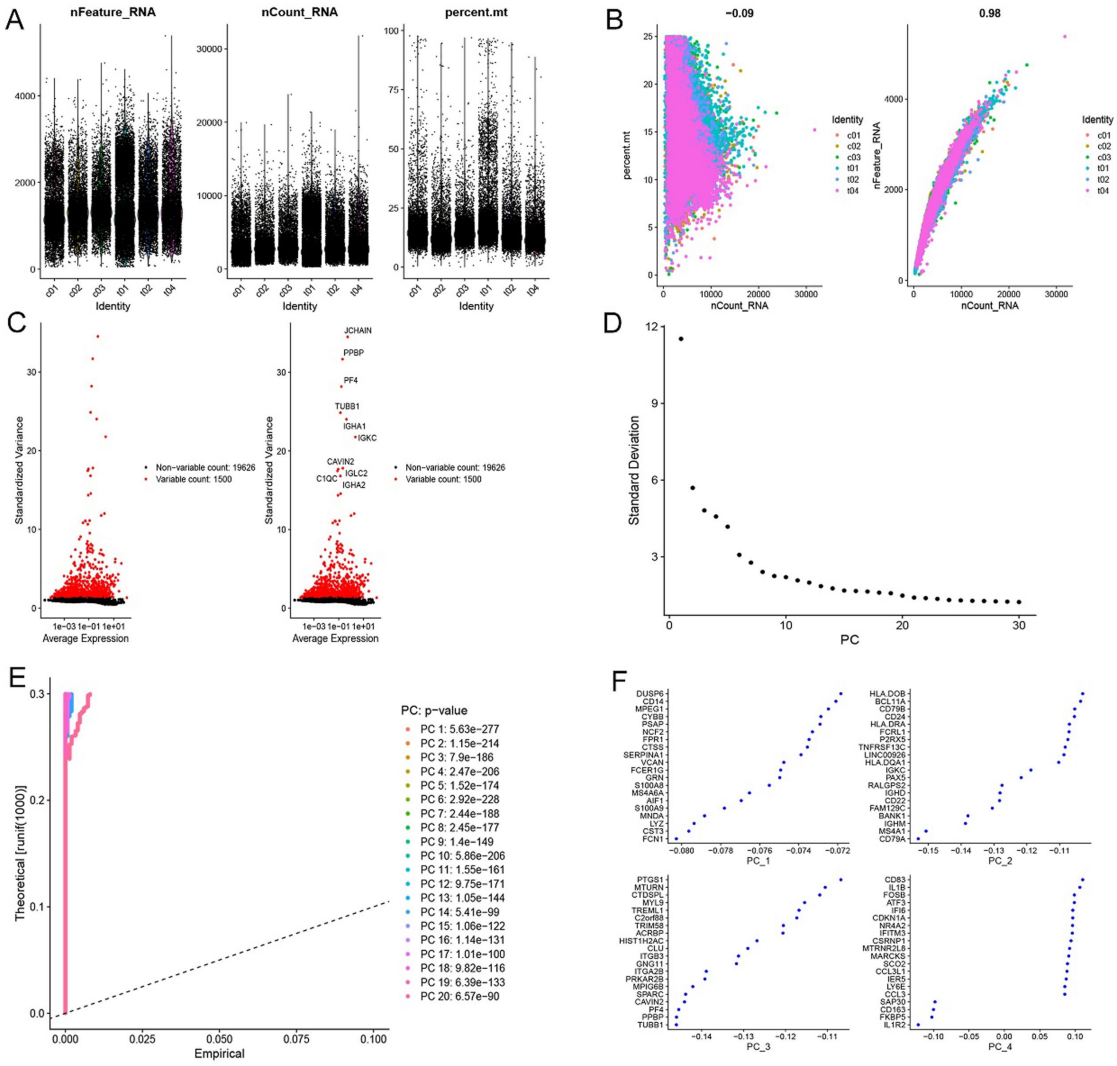
Single-cell RNA sequencing data preprocessing and quality control. **(A)** Quality control filtering metrics (genes detected per cell, cells expressing each gene, mitochondrial transcript proportion). **(B)** Quality control correlation scatter plots of single-cell RNA sequencing (mitochondrial genes vs. total reads, detected genes vs. total reads). **(C)** Scatter plot of gene expression variability (mean expression versus dispersion). **(D)** Elbow plot for determining the optimal number of principal components. **(E)** JackStraw permutation test results. **(F)** Scatter plot of gene expression across principal components.

We applied rigorous quality control to the raw single-cell transcriptomic data, resulting in 36,703 high-quality cells retained for downstream analyses (Figure 2A). Mitochondrial genes show a weak correlation with total reads, whereas detected genes exhibit a strong correlation with total reads (Figure 2B). Based on mean expression and standardized variance, 1,500 highly variable genes were identified (Figure 2C). Principal component analysis performed on these genes guided the selection of 10 principal components using the elbow method (Figure 2D). JackStraw permutation tests confirmed that the *p*-values for all top 10 principal components were significantly below the significance threshold (Figure 2E). Genes contributing most strongly to these 10 principal components were visualized. As illustrated for the first four PCs, each component was driven by a functionally coherent set of genes (Figure 2F).

**Figure 3:**
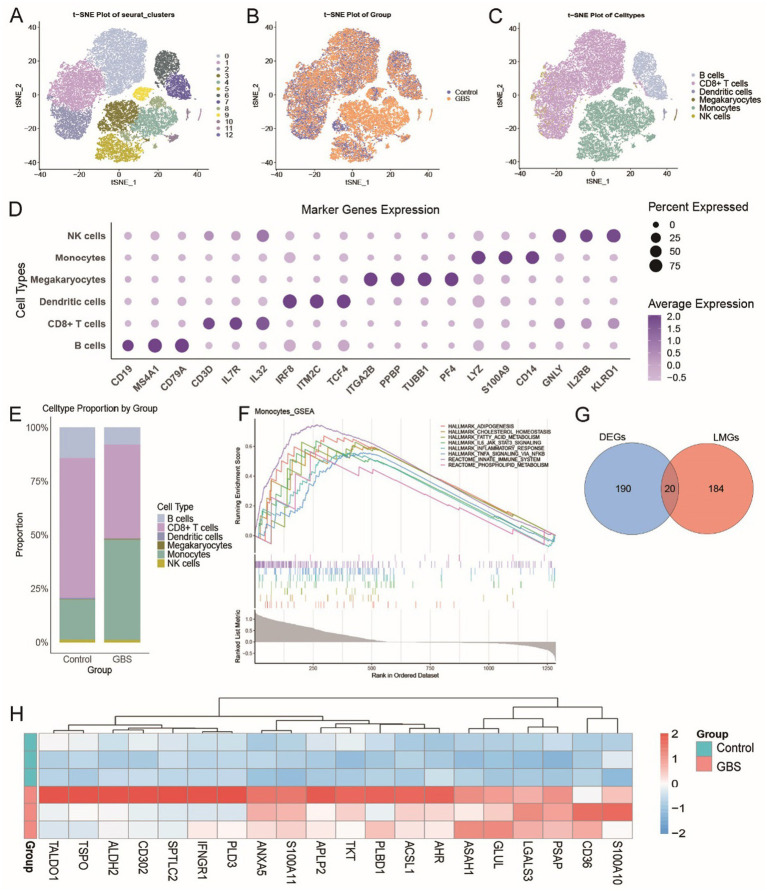
Peripheral blood monocyte profiles and lipid metabolism characteristics in GBS patients. **(A)** *t*-SNE plot showing 13 cell clusters identified by Seurat analysis. **(B)** *t*-SNE plot displaying cells from controls and GBS patients. **(C)** *t*-SNE visualization of the six annotated monocyte subpopulations. **(D)** Bubble plot illustrating the top 3 marker genes for each subpopulation. **(E)** Stacked bar plot comparing the proportions of monocyte subpopulations between controls and GBS patients. **(F)** GSEA reveals enrichment of lipid metabolism and immune-inflammatory pathways in monocytes. **(G)** Venn diagram showing the overlap between differentially expressed genes in GBS patients vs. controls and lipid metabolism-related genes. **(H)** Heatmap of the expression of 20 overlapping genes in GBS patients and controls.

Through cell annotation, the cells were classified into six major types: B cells, CD8 + T cells, monocytes, NK cells, dendritic cells, and progenitors (Figures 3A–D). We observed that the proportion of monocytes in GBS patients (47%) was higher than in the control group (19%). Given the marked expansion of monocytes in GBS, we subsequently performed an in-depth analysis of this cell population (Figure 3E).

GSEA revealed that monocytes from GBS patients were enriched in lipid metabolism-related pathways (e.g., cholesterol homeostasis, fatty acid metabolism) and inflammatory pathways (e.g., TNF-*α* signaling via NF-κB, innate immune system) (Figure 3F). To further identify the key genes regulating these phenotypes, we intersected the DEGs in monocytes from GBS patients and controls with lipid metabolism-related gene sets, resulting in 20 overlapping genes (e.g., ACSL1, AHR, CD36, S100A10, S100A11, TSPO) (Figure 3G). Heatmaps showed that these genes were upregulated in GBS patients (Figure 3H).

### 3.3 Altered monocyte subset composition and functional heterogeneity in GBS patients

**Figure 4:**
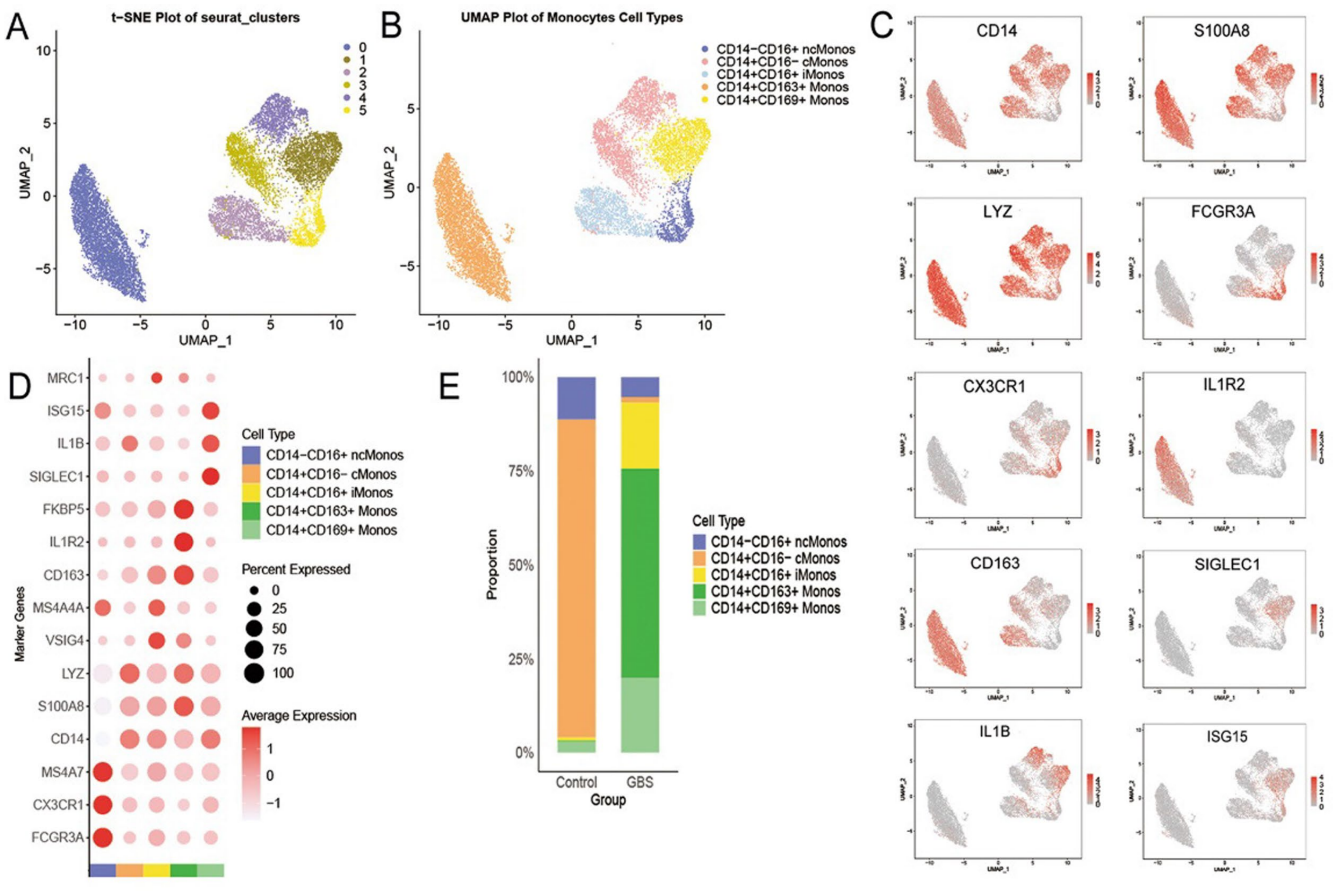
Identification and proportion analysis of monocyte subsets. **(A)** UMAP clustering plot of monocytes. **(B)** UMAP annotated plot of monocyte subsets. **(C)** UMAP expression feature plots of marker genes in monocytes. **(D)** Dot plot of marker genes for each monocyte subset. **(E)** Stacked bar chart showing the relative proportions of monocyte subsets in controls and GBS patients.

We reanalyzed 11,897 monocytes and classified them into five subpopulations based on marker gene expression, including CD14^+^CD16^−^ cMonos, CD14^+^CD16^+^ iMonos, CD14^−^CD16^+^ ncMonos, CD14^+^CD163^+^ Monos and CD14^+^CD169^+^ Monos (Figures 4A–D). The proportions of these monocyte subpopulations differed significantly between GBS patients and controls (Figure 4E). In the controls, CD14^+^CD16^−^ cMonos predominated (85%). In contrast, CD14^+^CD163^+^ Monos became the most abundant subset in GBS patients (56%), while the proportion of CD14^+^CD16^−^ cMonos dropped sharply to 1%. Concurrently, GBS patients exhibited increased proportions of CD14^+^CD16^+^ iMonos (18%) and CD14^+^CD169^+^ Monos (20%) compared to controls (1 and 3%, respectively), whereas the proportion of CD14^−^CD16^+^ ncMonos was slightly reduced (5% vs. 11% in controls).

**Figure 5:**
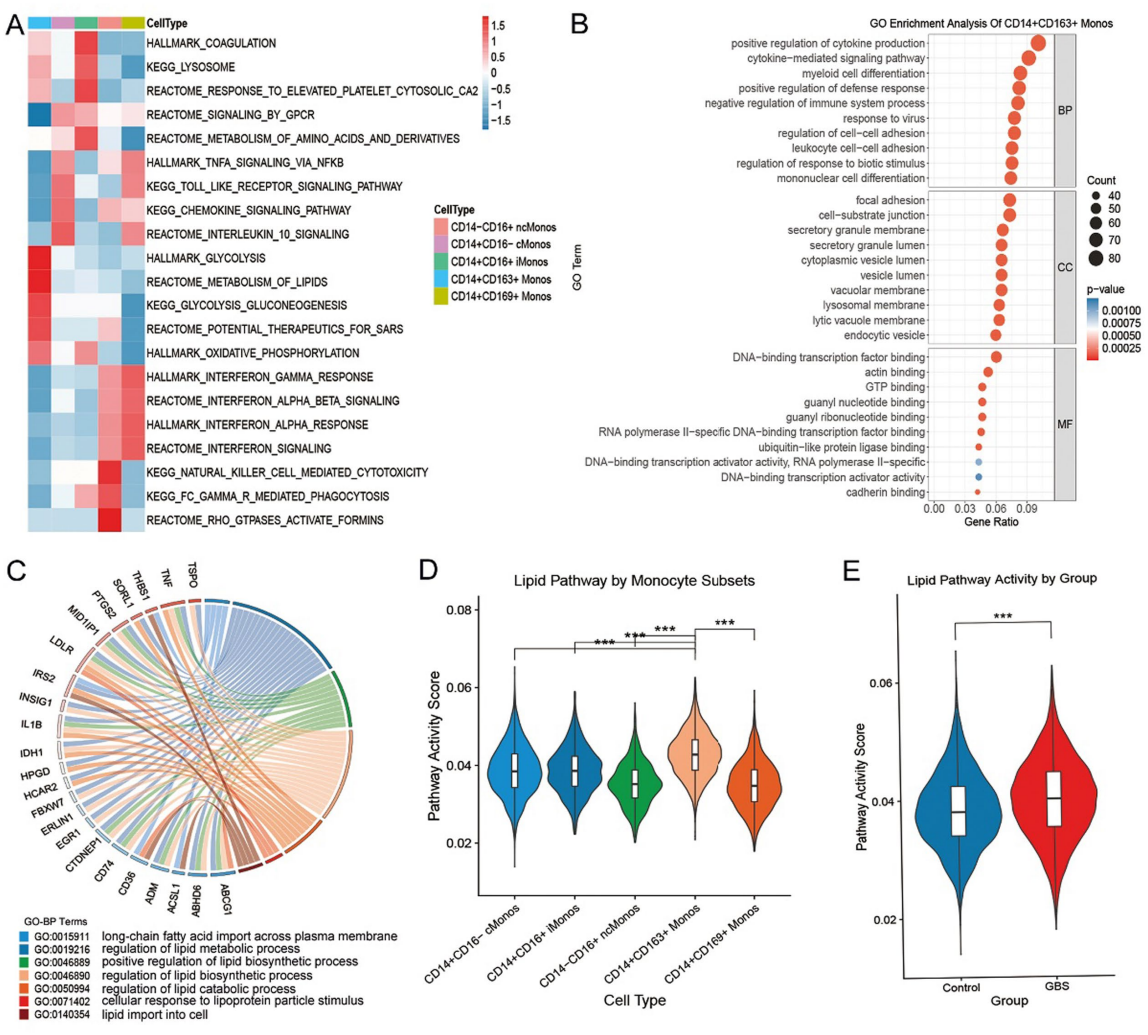
Functional enrichment and pathway activity analysis of monocyte subpopulations. **(A)** GSEA pathway enrichment heatmap for different monocyte subpopulations, with color gradients representing enrichment levels. **(B)** GO term bubble plot for CD14^+^CD163^+^ Monos. **(C)** String diagram of lipid metabolism-related GO terms and core genes in CD14^+^CD163^+^ Monos. **(D)** Violin plot of lipid metabolism pathway activity scores across monocyte subpopulations. **(E)** Violin plot comparing lipid metabolism pathway activity scores between controls and GBS patients. *** *p* < 0.001.

Functional enrichment analysis revealed specificity among different monocyte subpopulations. CD14^+^CD16^−^ cMonos are enriched in pathways such as interleukin-10 signaling and chemokine signaling. CD14^+^CD16^+^ iMonos are enriched in pathways like coagulation and response to elevated platelet cytosolic Ca^2+^. In contrast, CD14^−^CD16^+^ ncMonos show enrichment in pathways including natural killer cell–mediated cytotoxicity and Rho GTPase activated formins. Notably, the expanded CD14^+^CD163^+^ Monos in GBS patients is specifically enriched in glycolysis and lipid metabolism pathways, while the CD14^+^CD169^+^ Monos is highly enriched in interferon-gamma response and interferon signaling pathways (Figure 5A). GO analysis of the CD14^+^CD163^+^ Monos further confirmed its association with positive regulation of cytokine production, focal adhesion, and DNA − binding transcription factor binding (Figure 5B). A string diagram illustrates the association between lipid metabolism-related GO terms and their corresponding core genes (Figure 5C).

**Figure 6:**
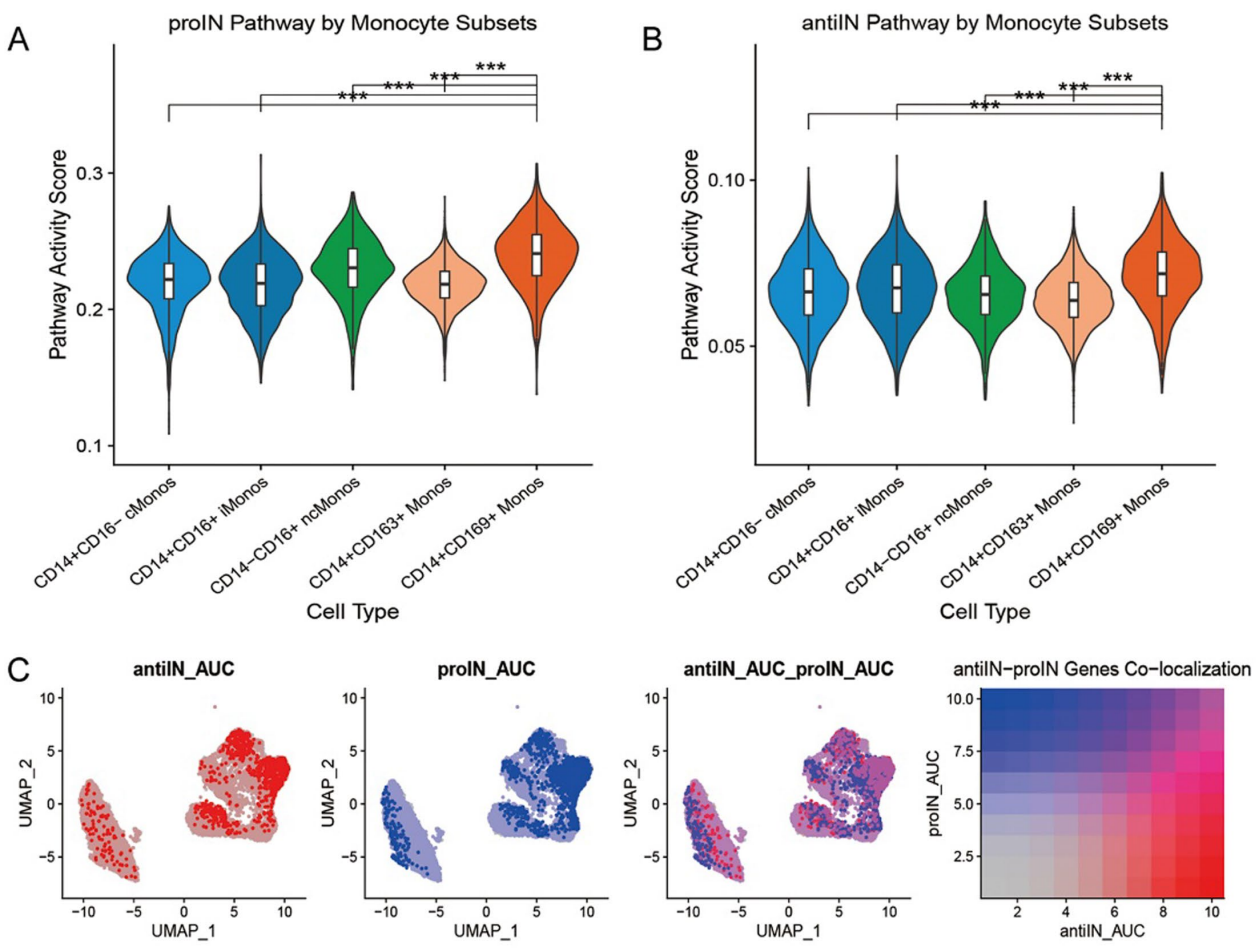
Analysis of pro-inflammatory and anti-inflammatory pathway activity in monocyte subpopulations. **(A)** Violin plots showing pro-inflammatory pathway activity scores across monocyte subpopulations. **(B)** Violin plots showing anti-inflammatory pathway activity scores across monocyte subpopulations. **(C)** UMAP visualization and colocalization analysis of pro-inflammatory and anti-inflammatory pathway activities in monocytes: the left and middle panels represent cell distributions in UMAP space for anti-inflammatory and pro-inflammatory pathway activities, respectively; the right panel shows a colocalization heatmap between the two pathway activities, with color gradient indicating colocalization intensity. *** *p* < 0.001.

Pathway activity scores quantified these functional differences: CD14^+^CD163^+^ Monos exhibited the highest lipid metabolism pathway activity among all subpopulations (Figure 5D), and this activity was significantly elevated in all monocytes from the GBS patients compared to controls (Figure 5E). Additionally, violin plots and UMAP visualizations of pathway activity revealed that both pro-inflammatory and anti-inflammatory pathway activities were highest in the CD14^+^CD169^+^ Monos (Figures 6A–C).

### 3.4 Directional differentiation of monocytes toward lipid metabolism reprogramming phenotypes

**Figure 7:**
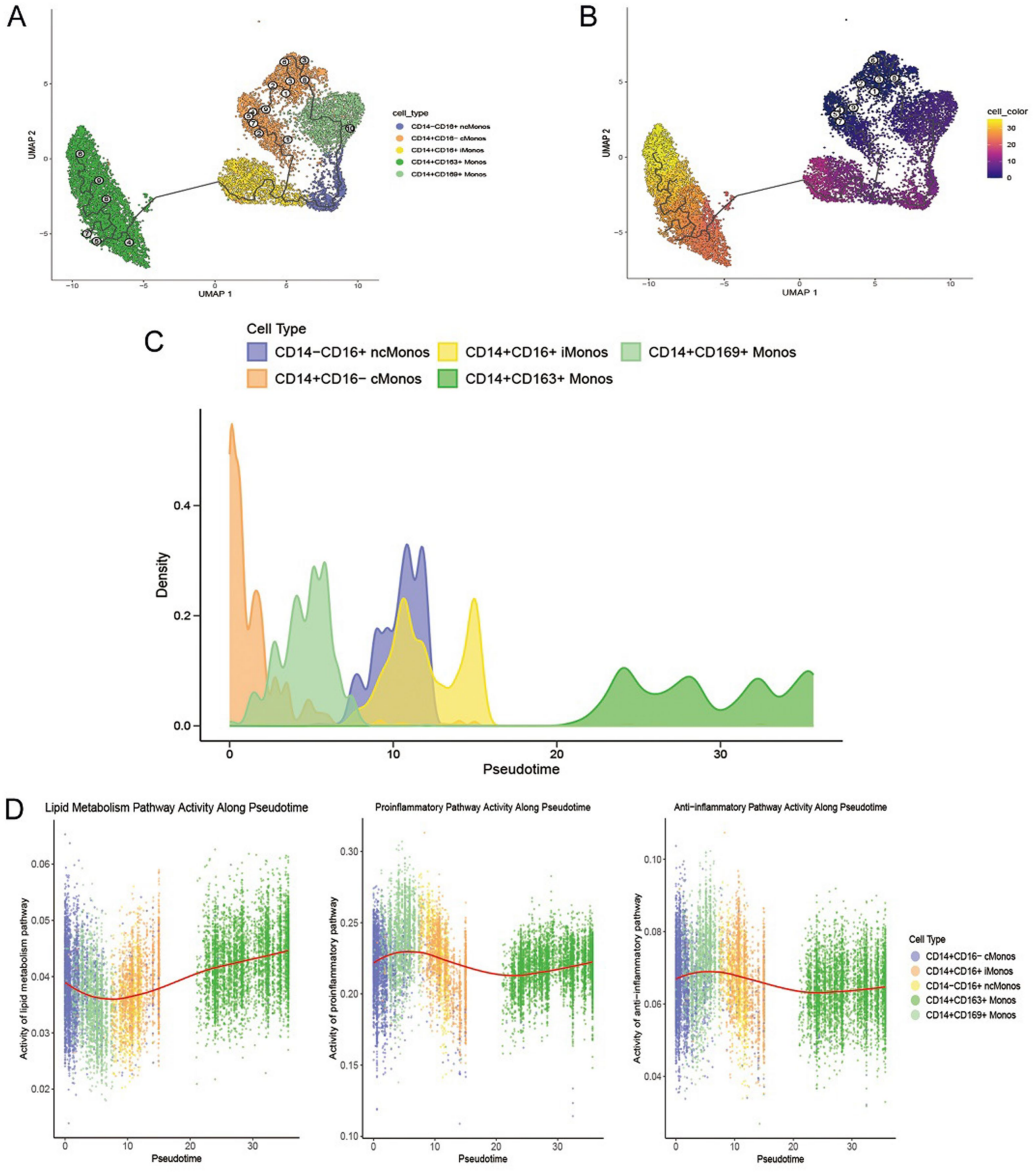
Pseudotime trajectories and functional dynamics of monocytes. **(A)** UMAP projection of five monocyte subpopulations. **(B)** UMAP projection of monocyte pseudotime values: color gradient corresponds to pseudotime value, showing the temporal progression along the pseudotime axis. **(C)** Pseudotime distribution density plots for each monocyte subpopulation. **(D)** Temporal dynamics of pathway activity over pseudotime: the left, middle, and right panels show the trends of lipid metabolism, pro-inflammatory, and anti-inflammatory pathway activities over pseudotime, respectively. Scatter points represent individual cell activity values, and the red curve indicates the overall trend line of pathway activity.

To delineate the developmental hierarchy among monocyte subsets, pseudotime trajectory was conducted. The results demonstrate that monocytes originate from CD14^+^CD16^−^ cMonos, subsequently pass through CD14^+^CD169^+^ Monos and CD14^+^CD16^+^ iMonos, and ultimately progress toward CD14^+^CD163^+^ Monos (Figures 7A–C).

Along the pseudotime trajectory, lipid metabolism pathway activity exhibited a transient decline during the CD14^+^CD169^+^ Monos stage, followed by a marked increase culminating in a peak in terminal CD14^+^CD163^+^ Monos. Meanwhile, both pro-inflammatory and anti-inflammatory pathway activities peaked during the CD14^+^CD169^+^ Monos stage, subsequently decreased through intermediate differentiation stages, and showed partial reactivation in terminal CD14^+^CD163^+^ Monos (Figure 7D).

### 3.5 CEBPB is a core regulator of lipid metabolism and differentiation

To identify core transcription factors regulating monocyte phenotypes in GBS, this study performed differential expression analysis on monocytes from patients and healthy controls. Based on the screening criteria, a total of 305 DEGs were identified, including CD163, FKBP5, HLA-C, HLA-DRB5, IL1R2, and PHC2.

**Figure 8:**
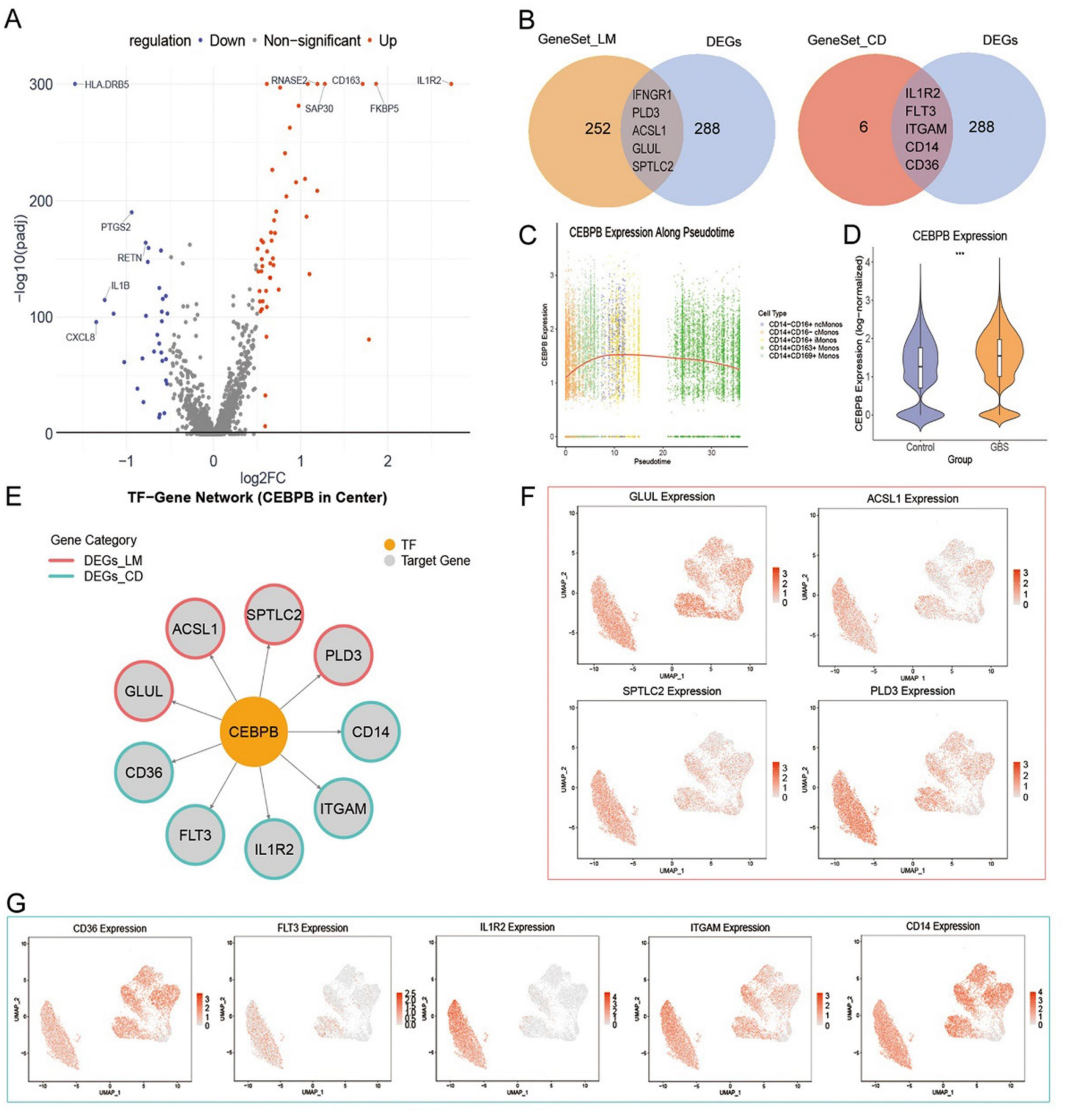
Transcription factor regulatory network analysis. **(A)** Volcano plot of DEGs in monocytes. **(B)** Venn diagram showing intersections between DEGs and functional gene sets. Left: DEGs and LM gene set; Right: DEGs and CD gene set. **(C)** Pseudotime trajectories expression dynamics of CEBPB. **(D)** Violin plot of CEBPB expression across groups. **(E)** Regulatory network of transcription factor-target interactions. Orange nodes: transcription factors; gray nodes: target genes, further annotated as LM-related (red) or CD-related (green). **(F)** UMAP projection of LM-related target genes expression. **(G)** UMAP projection of CD-related target genes expression. *** *p* < 0.001.

To focus on gene modules closely related to the functional phenotypes of monocytes in GBS, DEGs were intersected with gene sets associated with LM and CD. The analysis revealed five overlapping genes between the DEGs and the LM gene set, namely IFNGR1, PLD3, ACSL1, GLUL, and SPTLC2 (Figure 8A). Additionally, five genes overlapped with the CD gene set, including IL1R2, FLT3, ITGAM, CD14, and CD36 (Figure 8B). These two subsets were subsequently merged to construct the final target gene set.

Using the Cistrome Data Browser, we predicted potential upstream TFs for each gene in the target set, retaining the top 10 candidate TFs per gene. Based on occurrence frequency analysis, we selected the most recurrent differentially expressed TFs that collectively covered over 60% of target genes. Through this approach, CEBPB was identified as the core regulatory transcription factor.

Pseudotime trajectories indicated that CEBPB expression initially increased as monocytes differentiated toward the CD14^+^CD163^+^ Monos subset, reached its peak in the CD14^+^CD169^+^ Monos subpopulation, and subsequently declined slightly (Figure 8C). Comparative analysis further demonstrated that CEBPB expression was significantly elevated in monocytes from GBS patients compared with healthy controls (Figure 8D). Building on this finding, we constructed a transcriptional regulatory network centered on CEBPB (Figure 8E). Moreover, within the UMAP dimensionality reduction space, these target genes exhibited strong co-localization and were specifically enriched in the disease-associated CD14^+^CD163^+^ Monos subset (Figures 8F,G).

### 3.6 GEO dataset validation of monocyte features and core pathways

**Figure 9:**
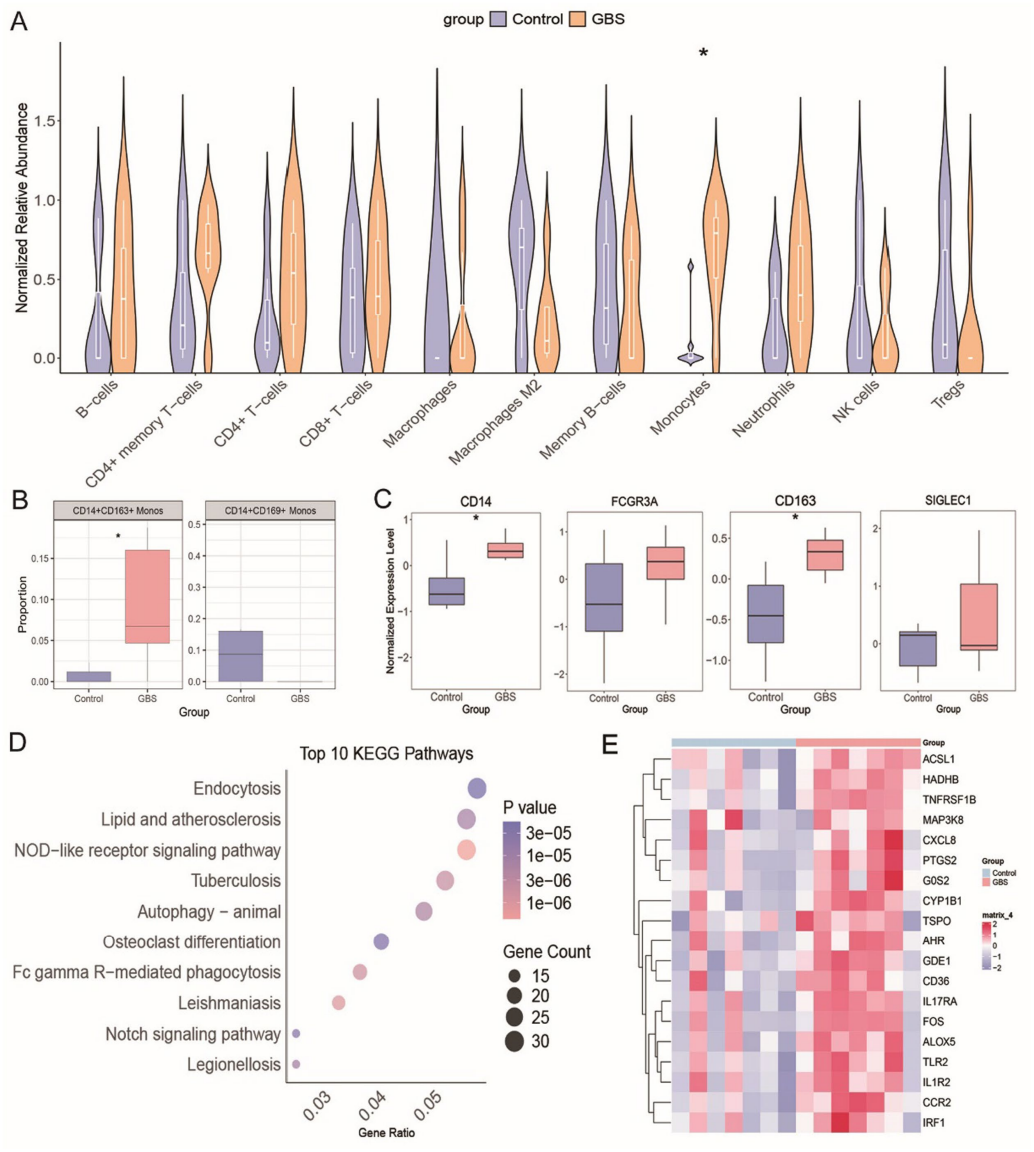
Independent dataset validation. **(A)** Violin plots showing relative abundance of different immune cell types in controls (blue) versus GBS patients (orange). **(B)** Bar plots showing the estimated proportions of CD14^+^CD163^+^ Monos and CD14^+^CD169^+^ Monos subpopulations derived from bulk RNA-seq data via deconvolution analysis in controls versus GBS patients. **(C)** Box plots of expression levels of monocyte marker genes (CD14, FCGR3A, CD163, SIGLEC1) in controls versus GBS patients. **(D)** Bubble plot of the top 10 KEGG pathways significantly enriched in DEGs. **(E)** Heatmap of lipid metabolism and pro-inflammatory gene expression levels in controls versus GBS patients, with color gradient (blue to red) indicating low to high gene expression. * *p* < 0.05.

To validate these findings, we analyzed an independent peripheral blood bulk RNA-seq dataset (GSE31014). Cell deconvolution confirmed a significantly higher relative abundance of monocytes in the peripheral blood of GBS patients compared to healthy controls (Figure 9A). Further deconvolution with CIBERSORTx, utilizing our scRNA-seq-derived signature matrix, demonstrated a significant expansion of the CD14^+^CD163^+^ Monos subset. In contrast, the CD14^+^CD169^+^ Monos subset exhibited no significant change (Figure 9B). Monocytes marker genes CD14and CD163showed overall significant upregulation in the GBS patients, while the expression of FCGR3A(CD16) and SIGLEC1(CD169) remained unchanged (Figure 9C). KEGG pathway enrichment analysis again revealed significant enrichment of differentially expressed genes in the lipid and atherosclerosis, NOD-like receptor signaling pathways in the GBS patients (Figure 9D). Heatmaps revealed widespread upregulation of key genes involved in lipid metabolism and proinflammation in the GBS patients (Figure 9E).

## 4 Discussion

This study combined clinical retrospective analysis with single-cell analysis to investigate subpopulation shifts, metabolic reprogramming, and associated transcriptional regulatory networks in peripheral blood mononuclear cells of GBS patients. The results revealed a distinct lipid-immune correlation pattern in GBS patients, characterized by elevated TG, VLDL and RC levels, alongside reduced HDL and APOA1 levels, together with increased MONO. Multivariate logistic regression analyses further confirmed that these parameters were independent predictors significantly associated with GBS. Single-cell analysis further showed marked remodeling of monocyte subpopulations in GBS patients, primarily reflecting expansion of the CD14^+^CD163^+^ Monos subset and enhanced lipid metabolic activity. Pseudotime trajectory suggested monocyte differentiation toward a lipid metabolism-reprogrammed phenotype, with CEBPB potentially playing a central regulatory role in this process. These findings provide new insights into the immunemetabolic mechanisms of GBS and lay a foundation for identifying potential therapeutic targets.

Our findings indicate that alcohol consumption and diabetes may be independent risk factors for GBS. Alcohol can lead to multiple neurological impairments, including peripheral neuropathy, cerebellar degeneration, cognitive dysfunction, and autonomic dysfunction. These effects result from both direct neurotoxicity and secondary nutritional deficiencies (23, 24). Diabetes is a frequent cause of peripheral nerve damage. This damage primarily manifests as distal symmetric sensory-motor axonal neuropathy, resulting from multiple mechanisms such as hyperglycemia-induced metabolic disorders, oxidative stress, inflammatory responses, and microvascular injury (25). Furthermore, this study found elevated levels of WBC, NEUT, and MONO in GBS patients, accompanied by decreased LYMPH, collectively presenting a typical acute inflammatory response state. The marked elevation of monocytes warrants particular attention. Recent studies indicate that monocytes are not merely precursor cells for macrophages but represent a highly functionally heterogeneous group of immune cells. In autoimmune diseases, specific inflammatory monocyte subsets migrate to target tissues, directly participating in pathological processes by secreting proinflammatory factors and presenting self-antigens, thereby driving disease progression (26). Consistent with prior findings, we observed elevated TG, LDL, and RC levels, alongside reduced HDL and APOA1 levels, in patients with GBS (16). The dyslipidemia profile of high VLDL/TG and low HDL, observed in GBS, warrants further investigation as it is also observed in multiple sclerosis (27). Plasma apolipoprotein A1 (APOA1), as the core structural protein of HDL, exerts multifaceted anti-inflammatory effects: Firstly, its levels negatively correlate with the expression of pro-inflammatory recruitment markers on monocyte surfaces (e.g., CD11b, CD11c, CD29, and CCR2), suggesting it suppresses monocyte activation and migration (14); secondly, it downregulates expression of endothelial cell adhesion molecules (e.g., E-selectin, ICAM-1, VCAM-1), reduces release of proinflammatory factors like TNF-*α* and IL-6, and blocks inflammatory signaling pathways triggered by LPS or viral NS1 protein, thereby protecting vascular endothelium and exerting systemic anti-inflammatory effects (28). Notably, this study found no significant difference in APOB levels between GBS patients and healthy controls, which diverges from the findings of some prior research (15, 16). This discrepancy may be attributed to several factors. First, the present study included only classical GBS, which may differ in terms of systemic inflammatory intensity and metabolic disturbance. Second, regional or genetic variations might also affect lipoprotein synthesis and metabolic regulation. Therefore, future studies should perform stratified analyses based on GBS clinical subtypes within a standardized sampling timeframe to further clarify the role of APOB in GBS.

Monocytes, as core effector cells in peripheral immune responses, play a pivotal role in peripheral nerve inflammation. Studies indicate that following peripheral nerve injury, large numbers of circulating monocytes migrate into nerve tissue through transendothelial migration and subsequently differentiate locally into macrophages (29). Under conditions of high glucose and dyslipidemia (such as oxLDL and VLDL), the downregulation of Sestrin2 in monocytes leads to AMPK inhibition and mTOR overactivation, driving their polarization toward the pro-inflammatory M1 phenotype and enhancing adhesion and foam cell formation. This reveals the mechanism by which dyslipidemia activates monocytes through the metabolism-inflammation axis (30). Elevated RC upregulates the expression of TLR2/4 and NF-κB in monocytes and enhances LPS-induced IL-1β release, indicating its role in promoting the onset of GBS through activation of innate immune pathways (16). We found that CD14^+^CD163^+^ Monos were significantly expanded in GBS, primarily enriched in glycolysis and lipid metabolism pathways. Based on the known functions of CD163 (a scavenger receptor involved in lipid clearance and anti-inflammatory repair), it is hypothesized that this subset may play a metabolic adaptive role in GBS by enhancing lipid metabolism to provide energy for immune cells and clearing apoptotic debris generated by neuroinflammatory damage, thereby exerting a compensatory neuroprotective effect (30, 31). The proportion of CD14^+^CD169^+^ Monos in GBS is also significantly increased, and they are enriched in pathways such as interferon-gamma response and interferon signaling pathways. Based on the established role of CD169 in viral recognition and antigen presentation, as well as its facilitation of interactions between monocytes and T cells (32), and given the pathological context of GBS often occurring secondary to respiratory or gastrointestinal infections (33), we hypothesize that this subset may represent an inflammatory-initiating population in GBS.

Pseudotime trajectorie analysis revealed the differentiation trajectory of peripheral blood mononuclear cells in GBS patients, accompanied by dynamic functional changes: pro-inflammatory activity initially increased and then declined, while lipid metabolism activity gradually strengthened. This aligns with the functional transition pattern of immune cells shifting from stress response to injury repair. A similar functional switch has been observed previously in macrophages during infection responses (34). Excessive activation of the TNF-*α*/NF-κB pathway in the CD14^+^CD169^+^ Monos subpopulation leads to the release of TNF-α and IL-6, which in turn may aggravate Schwann cell damage and demyelination. This pathogenic mechanism has been confirmed in studies of sciatic nerve injury using an experimental autoimmune neuritis model (35). The CD14^+^CD163^+^ Monos subpopulation exhibits significantly enhanced lipid metabolism activity. GO analysis indicates its functional enrichment in lipid transport, long-chain fatty acid transmembrane transport, and lipoprotein particle response. Previous studies have indirectly supported the association between this cell subset and abnormal lipid metabolism. Specifically, among elderly HIV-positive males, the activation level of CD163^+^ macrophages showed a positive correlation with serum TG levels and a negative correlation with protective HDL-related lipid components. Gene set enrichment analysis further indicated that lipid metabolism pathways in CD163^+^ cells were significantly associated with multiple dyslipidemia parameters (36). Additionally, in kidney transplant recipients with chronic inflammatory states, elevated levels of soluble CD163 were positively correlated with increased TG, decreased HDL, and overall metabolic dysregulation (37). These observations suggest that in inflammatory pathological states, elevated TG levels and decreased HDL function may influence monocyte differentiation into the CD163^+^ subtype by reprogramming lipid metabolism. This aligns closely with the findings of the present study, which indicate enhanced differentiation of classical monocytes into the CD14^+^CD163^+^ Monos subtype accompanied by abnormal TG/HDL levels. Together, these results provide cross-disease insights into the potential link between these metabolic and immunological processes. However, it is essential to emphasize that dyslipidemia is driven by multiple factors, including genetics, metabolic homeostasis, and inflammatory stress. The enhanced activation of lipid metabolism in the CD14^+^CD163^+^ Monos subset is more likely an adaptive response to the pathological microenvironment of GBS. Similar metabolic adaptation mechanisms have been described in tumor-associated macrophages (38). Nevertheless, the direct causal relationship and relevant regulatory targets require further validation through cell functional experiments, such as targeted inhibition of lipid metabolism pathways in this subset.

We found that in peripheral blood monocytes from GBS patients, the core transcription factor CEBPB may regulates the expression of PLD3, SPTLC2, ACSL1, GLUL, CD36, FLT3, IL1R1, ITGAM, and CD14, which may affect lipid metabolism and cell differentiation processes. CEBPB appears to exhibit dual roles in lipid metabolism and inflammatory responses. During early lipogenesis, it activates and cooperates with other factors to regulate autophagy-related genes (e.g., Becn1, Map1lc3b), thereby promoting the differentiation of preadipocytes into mature adipocytes and facilitating lipid storage and adipocyte metabolic reprogramming (39). Conversely, in contexts such as vascular endothelium, CEBPB significantly upregulates proinflammatory factors like IL-6, which drives local inflammatory responses and facilitates the infiltration of monocytes/macrophages, underpinning its key proinflammatory role in a variety of diseases. Additionally, CEBPB serves as a key transcription factor in the differentiation of granulocyte-monocyte progenitor cells into monocytes (40). Collectively, these findings provide important molecular insights into the multifaceted functions of CEBPB and offer potential explanations for the immunometabolic dysregulation observed in GBS.

Results validated using an independent GSE31014 bulk RNA-seq cohort corroborate our single-cell findings by confirming a concurrent expansion of the total monocyte population and the CD14^+^CD163^+^ Monos subset in GBS patients. In contrast, the lack of significant SIGLEC1 (encoding CD169) upregulation in this bulk dataset does not contradict the expansion of the CD14^+^CD169^+^ Monos subset observed in scRNA-seq; rather, it reflects a dilution effect where the transcriptional signal of the expanding CD14^+^CD169^+^ Monos population is obscured by its substantially lower expansion magnitude relative to the dominantly amplified CD14^+^CD163^+^ Monos subset. The widespread overexpression of key lipid metabolism and proinflammatory genes further supports the co-activation of immune and metabolic pathways in GBS.

This study presents single-cell transcriptomic evidence for dynamic reprogramming of peripheral blood monocytes in GBS patients, characterized by a shift from inflammatory to metabolic phenotypes. CEBPB was identified as a central regulatory hub potentially involved in this process. The changes observed in specific monocyte subsets, together with characteristic lipid abnormalities such as elevated triglycerides and reduced high-density lipoprotein cholesterol, form an easily accessible set of non-invasive biomarkers. Moreover, CEBPB and its predicted downstream effectors, including ACSL1 and CD36, represent potential targets for immunometabolic therapeutic strategies. However, the study has several limitations. First, our scRNA-seq analysis was based on a small cohort (*n* = 3 GBS patients and *n* = 3 controls). We observed drastic, cliff-like shifts in monocyte subpopulations, specifically a sharp decline in classical monocytes and a concurrent expansion of the CD14^+^CD163^+^ Monos subset. Given the limited sample size, these extreme proportional changes are vulnerable to potential batch effects or individual patient anomalies and may not adequately reflect the heterogeneity of the broader GBS population. Consequently, the lack of flow cytometric validation in a larger independent cohort currently precludes us from definitively ruling out technical artifacts or confirming the precise magnitude of this subset remodeling at the individual level. Second, without stratification by disease stage, it remains unclear whether changes in monocyte subsets and their lipid metabolic activity follow a stage-dependent pattern. Furthermore, the proposed regulatory role of CEBPB is primarily based on bioinformatic inference and has not yet been confirmed by functional *in vitro* experiments. Therefore, future studies should incorporate flow cytometric validation of key monocyte subsets, conduct in vitro functional assays such as CEBPB perturbation experiments, and utilize animal models to mechanistically verify the roles of core molecules and bolster the translational relevance of these findings.

## 5 Conclusion

Our study reveals synergistic alterations in dyslipidemia and monocyte remodeling in GBS patients, where lipid metabolism reprogramming characterizing the CD14^+^CD163^+^ Monos subset and proinflammatory phenotypes associated with the CD14^+^CD169^+^ Monos subset constitute core pathological features. As a key regulator, CEBPB coordinates lipid metabolism and differentiation-related gene networks to potentially promote monocyte phenotypic conversion, providing molecular targets for precision diagnosis and treatment of GBS.

## Data Availability

Publicly available datasets were analyzed in this study. This data can be found at: the scRNA-seq data are available in the National Omics Data Encyclopedia (NODE) repository, under project accession numbers OEP002315 and OEP002701 (https://www.biosino.org/node/), and in the Gene Expression Omnibus (GEO) repository under accession number GSE31014.

## Footnotes

1
https://db3.cistrome.org/browser/

2
https://comphealth.ucsf.edu/app/xcell

## Glossary

APOA1: Apolipoprotein A1
APOB: Apolipoprotein B
BP: Biological processes
CC: Cellular Component
CD: Cell differentiation
CHOL: Total cholesterol
DEGs: Differentially expressed genes
GBS: Guillain–Barré syndrome
GO: Gene Ontology
GSEA: Gene set enrichment analysis
HDL: High-density lipoprotein
KEGG: Kyoto Encyclopedia of Genes and Genomes
LDL: Low-density lipoprotein cholesterol
LM: Lipid metabolism
Lpa: Lipoprotein(a)
LYMPH: Lymphocyte count
MF: Molecular Function
MONO: Monocyte count
NEUT: Neutrophil count
RC: Residual cholesterol
scRNA-seq: Single-cell RNA sequencing
TG: Triglycerides
VLDL: Very low-density lipoprotein
WBC: White blood cell count

## References

[1] McGrogan A Madle GC Seaman HE de Vries CS. The epidemiology of Guillain-Barré syndrome worldwide. Neuroepidemiology. (2009) 32:150–63. doi: 10.1159/000184748 19088488

[2] Zheng P Tian D-C Xiu Y Wang Y Shi F-D. Incidence of Guillain-Barré syndrome (GBS) in China: a national population-based study. Lancet Reg Health West Pac. (2022) 18:100302. doi: 10.1016/j.lanwpc.2021.100302, 35024648 PMC8661041

[3] Leonhard SE Papri N Querol L Rinaldi S Shahrizaila N Jacobs BC. Guillain–Barré syndrome. Nat Rev Dis Primers. (2024) 10:97. doi: 10.1038/s41572-024-00580-4, 39702645

[4] Fokke C van den Berg B Drenthen J Walgaard C van Doorn PA Jacobs BC. Diagnosis of Guillain-Barre syndrome and validation of Brighton criteria. Brain. (2013) 137:33–43. doi: 10.1093/brain/awt285, 24163275

[5] Vucic S Cairns KD Black KR Tick Chong PS Cros D. Neurophysiologic findings in early acute inflammatory demyelinating polyradiculoneuropathy. Clin Neurophysiol. (2004) 115:2329–35. doi: 10.1016/j.clinph.2004.05.009, 15351375

[6] Willison HJ. Anti-ganglioside antibodies in peripheral nerve pathology. Methods Mol Biol. (2018) 1804:173–88. doi: 10.1007/978-1-4939-8552-4_7 29926408

[7] Thomma RCM Fokke C Walgaard C Vermeulen-de Jongh DMC Tio-Gillen A van Rijs W . High and persistent anti-GM1 antibody titers are associated with poor clinical recovery in Guillain-Barré syndrome. Neurol Neuroimmunol Neuroinflamm. (2023) 10:e200107. doi: 10.1212/NXI.0000000000200107, 37059469 PMC10119811

[8] Doets AY Hughes RAC Brassington R Hadden RDM Pritchard J. Pharmacological treatment other than corticosteroids, intravenous immunoglobulin and plasma exchange for Guillain-Barré syndrome. Cochrane Database Syst Rev. (2020) 2020:CD008630. doi: 10.1002/14651858.CD008630.pub5 PMC6984651 31981368

[9] Doets AY Verboon C van den Berg B Harbo T Cornblath DR Willison HJ . Regional variation of Guillain-Barré syndrome. Brain. (2018) 141:2866–77. doi: 10.1093/brain/awy232, 30247567

[10] Ginhoux F Mildner A Gautier EL Schlitzer A Jakubzick C Varol C . Editorial: monocyte heterogeneity and function. Front Immunol. (2021) 11:626725. doi: 10.3389/fimmu.2020.626725, 33488633 PMC7817760

[11] Kraaijenhof JM Stroes ESG. Inflammatory effects of triglycerides. JACC Basic Transl Sci. (2023) 8:476–8. doi: 10.1016/j.jacbts.2023.04.005, 37325399 PMC10264562

[12] Stiekema LCA Willemsen L Kaiser Y Prange KHM Wareham NJ Boekholdt SM . Impact of cholesterol on proinflammatory monocyte production by the bone marrow. Eur Heart J. (2021) 42:4309–20. doi: 10.1093/eurheartj/ehab465, 34343254 PMC8572558

[13] Lee C Sigari F Segrado T Hörkkö S Hama S Subbaiah PV . All ApoB-containing lipoproteins induce monocyte chemotaxis and adhesion when minimally modified. Arterioscler Thromb Vasc Biol. (1999) 19:1437–46. doi: 10.1161/01.ATV.19.6.1437, 10364074

[14] Patel VK Williams H Li SCH Fletcher JP Medbury HJ. Monocyte subset recruitment marker profile is inversely associated with blood ApoA1 levels. Front Immunol. (2021) 12:616305. doi: 10.3389/fimmu.2021.616305, 33717107 PMC7952433

[15] Wang L Ding Y Liu J Zheng G Li S Jiang W . The analysis of serum lipids profile in Guillain-Barre syndrome. Front Immunol. (2023) 14:1301577. doi: 10.3389/fimmu.2023.1301577, 38143756 PMC10739405

[16] Ding Y Wang L Sun J Shi Y Li G Luan X . Remnant cholesterol and dyslipidemia are risk factors for Guillain–Barré syndrome and severe Guillain–Barré syndrome by promoting monocyte activation. Front Immunol. (2022) 13:946825. doi: 10.3389/fimmu.2022.946825, 35911688 PMC9326451

[17] Li M Song J Yin P Chen H Wang Y Xu C . Single-cell analysis reveals novel clonally expanded monocytes associated with IL1β–IL1R2 pair in acute inflammatory demyelinating polyneuropathy. Sci Rep. (2023) 13:5862. doi: 10.1038/s41598-023-32427-5, 37041166 PMC10088807

[18] Sekerkova A Krepsova E Brabcova E Slatinska J Viklicky O Lanska V . CD14+CD16+ and CD14+CD163+ monocyte subpopulations in kidney allograft transplantation. BMC Immunol. (2014) 15:4. doi: 10.1186/1471-2172-15-4, 24499053 PMC3918100

[19] Hou J Wang X Zhang M Wang M Gao P Jiang Y. Circulating CD14+CD163+CD209+ M2-like monocytes are associated with the severity of infection in *Helicobacter pylori*-positive patients. Mol Immunol. (2019) 108:13–22. doi: 10.1016/j.molimm.2019.01.017, 30771733

[20] Suzuki H Li C Luo X Lin Y Tang X Ling L . A higher frequency of CD14+CD169+ monocytes/macrophages in patients with colorectal cancer. PLoS One. (2015) 10:e0141817. doi: 10.1371/journal.pone.0141817 26509874 PMC4625021

[21] Affandi AJ Grabowska J Olesek K Lopez Venegas M Barbaria A Rodríguez E . Selective tumor antigen vaccine delivery to human CD169+ antigen-presenting cells using ganglioside-liposomes. Proc Natl Acad Sci. (2020) 117:27528–39. doi: 10.1073/pnas.2006186117, 33067394 PMC7959579

[22] Coillard A Segura E. In vivo differentiation of human monocytes. Front Immunol. (2019) 10:01907. doi: 10.3389/fimmu.2019.01907, 31456804 PMC6700358

[23] Julian T Glascow N Syeed R Zis P. Alcohol-related peripheral neuropathy: a systematic review and meta-analysis. J Neurol. (2018) 266:2907–19. doi: 10.1007/s00415-018-9123-1, 30467601 PMC6851213

[24] Hammoud N Jimenez-Shahed J. Chronic neurologic effects of alcohol. Clin Liver Dis. (2019) 23:141–55. doi: 10.1016/j.cld.2018.09.010, 30454828

[25] Galiero R Caturano A Vetrano E Beccia D Brin C Alfano M . Peripheral neuropathy in diabetes mellitus: Pathogenetic mechanisms and diagnostic options. Int J Mol Sci. (2023) 24:3554. doi: 10.3390/ijms24043554, 36834971 PMC9967934

[26] Guilliams M Mildner A Yona S. Developmental and functional heterogeneity of monocytes. Immunity. (2018) 49:595–613. doi: 10.1016/j.immuni.2018.10.005, 30332628

[27] Tettey P Simpson S Taylor B Blizzard L Ponsonby A-L Dwyer T . An adverse lipid profile is associated with disability and progression in disability, in people with MS. Mult Scler J. (2014) 20:1737–44. doi: 10.1177/1352458514533162, 24829292

[28] Guo K Hu C Li L Liu X Liu Y Zhang D . ApoA1/HDL and sepsis-associated vascular endothelial injury: a narrative review. Crit Care. (2025) 29:426. doi: 10.1186/s13054-025-05668-1, 41063278 PMC12506352

[29] Mueller M Leonhard C Wacker K Ringelstein EB Okabe M Hickey WF . Macrophage response to peripheral nerve injury: the quantitative contribution of resident and Hematogenous macrophages. Lab Investig. (2003) 83:175–85. doi: 10.1097/01.LAB.0000056993.28149.BF, 12594233

[30] Sundararajan S Jayachandran I Balasubramanyam M Mohan V Venkatesan B Manickam N. Sestrin2 regulates monocyte activation through AMPK-mTOR nexus under high-glucose and dyslipidemic conditions. J Cell Biochem. (2018) 120:8201–13. doi: 10.1002/jcb.28102, 30450765

[31] Govindappa PK Elfar JC. Erythropoietin promotes M2 macrophage phagocytosis of Schwann cells in peripheral nerve injury. Cell Death Dis. (2022) 13:245. doi: 10.1038/s41419-022-04671-6, 35296651 PMC8927417

[32] Affandi AJ Olesek K Grabowska J Nijen Twilhaar MK Rodríguez E Saris A . CD169 defines activated CD14+ monocytes with enhanced CD8+ T cell activation capacity. Front Immunol. (2021) 12:697840. doi: 10.3389/fimmu.2021.697840, 34394090 PMC8356644

[33] Shahrizaila N Lehmann HC Kuwabara S. Guillain-Barré syndrome. Lancet. (2021) 397:1214–28. doi: 10.1016/s0140-6736(21)00517-1, 33647239

[34] Locati M Curtale G Mantovani A. Diversity, mechanisms, and significance of macrophage plasticity. Annu Rev Pathol. (2020) 15:123–47. doi: 10.1146/annurev-pathmechdis-012418-012718, 31530089 PMC7176483

[35] Zhang Z Zhang ZY Fauser U Schluesener HJ. Valproic acid attenuates inflammation in experimental autoimmune neuritis. Cell Mol Life Sci. (2008) 65:4055–65. doi: 10.1007/s00018-008-8521-4, 18953683 PMC11131833

[36] Yeoh H-L Cheng AC Cherry CL Weir JM Meikle PJ Hoy JF . Immunometabolic and Lipidomic markers associated with the frailty index and quality of life in aging HIV+ men on antiretroviral therapy. EBioMedicine. (2017) 22:112–21. doi: 10.1016/j.ebiom.2017.07.015, 28754302 PMC5552224

[37] El Aggan H Mahmoud S El Shair H Elabd H. Increased macrophage activation marker soluble CD163 is associated with graft dysfunction and metabolic derangements in renal transplant recipients. Biom J. (2021) 44:S179–89. doi: 10.1016/j.bj.2020.09.004, 35300946 PMC9068521

[38] Vassiliou E Farias-Pereira R. Impact of lipid metabolism on macrophage polarization: implications for inflammation and tumor immunity. Int J Mol Sci. (2023) 24:12032. doi: 10.3390/ijms241512032, 37569407 PMC10418847

[39] Ahmed M Lai TH Hwang JS Zada S Pham TM Kim DR. Transcriptional regulation of autophagy genes via stage-specific activation of CEBPB and PPARG during Adipogenesis: a systematic study using public gene expression and transcription factor binding datasets. Cells. (2019) 8:1321. doi: 10.3390/cells8111321, 31731552 PMC6912425

[40] Zhang Z Bossila EA Li L Hu S Zhao Y. Central gene transcriptional regulatory networks shaping monocyte development in bone marrow. Front Immunol. (2022) 13:1011279. doi: 10.3389/fimmu.2022.1011279, 36304450 PMC9595600

